# Teaching Health Literacy and Digital Literacy Together at University Level: The FLOURISH Module

**DOI:** 10.1177/10901981231163609

**Published:** 2023-04-18

**Authors:** Alan F. Smeaton

**Affiliations:** 1 Dublin City University, Dublin, Ireland

**Keywords:** health literacy, data literacy, student wellness, personal data

## Abstract

Many universities have wellness programs to promote overall health and well-being. Using students’ own personal data as part of improving their own wellness would seem to be a natural fit given that most university students are already data and information literate. In this work, we aim to show how the interplay between health literacy and data literacy can be used and taught together. The method we use is the development and delivery of the FLOURISH module, an accredited, online-only but extra-curricular course that delivers practical tips in the areas that impact students’ everyday wellness including sleep, nutrition, work habits, procrastination, relationships with others, physical activity, positive psychology, critical thinking, and more. For most of these topics, students gather personal data related to the topic and submit an analysis of their data for assessment thus demonstrating how students can use their personal data for their benefit. More than 350 students have taken the module and an analysis of the use of online resources, as well as feedback on the module experience, are presented. The contributions of this article are to further endorse the need for health literacy and digital literacy for students, and we demonstrate that these can be taught together making each literacy more appealing to the digital natives of Generation Z who make up the majority of students. The implications for public health research and practice are that two student literacies, health and digital, are not independent and for our students, they should be taught together.

## Introduction

Many universities and colleges have wellness programs designed to promote the overall health and well-being of students and faculty. A nationwide survey of University programs in the United States which address student wellness identified 202 such programs, and that was 30 years ago, in 1992 (Dinger et al., 1992). A more recent critical review of the literature on college courses designed to improve wellness covered the years between 2000 and 2017 and found wellness or equivalent courses to be widespread at the university level. That work also found there is a lack of consensus on what exactly wellness is and what the courses should cover (Beauchemin et al., 2018).

The factors that are incorporated into wellness courses today can include physical, emotional, social, intellectual, and occupational wellness as described in Baldwin et al. (2017) and originally developed in Hettler’s original multifaceted model of holistic wellness (Hettler, 1984). Wellness courses often include a range of activities such as health screenings, fitness classes, stress management workshops, and other initiatives. Their goal is to help individuals develop healthy lifestyles and habits to improve their overall physical and mental health as well as improve their health literacy. In turn, this empowers individuals with the skills, knowledge, understanding, and confidence to understand and use health-related information and materials and to make informed decisions about their own lifestyle and behavior across the lifespan. The benefits of wellness courses in universities can be seen in a study reported by Ward et al. (2022), where students were found to have improved health literacy, healthier mindsets, healthier relationships with others and to have made positive changes to their lifestyles, for example regarding their exercise and nutrition.

In an attempt to understand students’ own priorities and perceptions regarding their health, one detailed study reported by Cass et al. (2021) found that stress and mental health are students’ primary concerns, more so than chronic or infectious diseases, although the data gathering for that study took place prior to the COVID-19 pandemic. The study also found that health was of secondary importance to overall happiness as far as students were concerned, although there is a strong interplay between the two. That study also identified stress, mental health, substance abuse, and nutrition as being students’ primary health concerns.

In 2019, the Union of Students in Ireland published a National Report on Student Mental Health in Third-Level Education in Ireland which included a survey of more than 3,300 students, and it found that one-third of students have clinically relevant levels of stress, anxiety, and depression (Price et al., 2019). In January 2021, Dublin City University Students’ Union reported a survey of 1,847 students where 81% felt alone more often than not while in lockdown, (only) 60% knew who to turn to if mental health affected their academic performance, and 53% found their workload unmanageable (Union, 2021). These are just two of many reports indicating a major problem facing our students as they navigate the stressful transition into juggling academic pressure from rigorous courses, managing their finances and perhaps having to take part-time or even full-time jobs while studying, worrying about their future and their career, their relationships with others, and separation from family (Mueller & Perreault, 2019). Although they are not explicitly called out as Generation Z in any of this literature, the majority of this current cohort of students are digital natives having grown up with the internet, social media, and the use of digital technologies.

Many, or even most, of our educational institutions focus on student, and faculty, wellness, well-being, health literacy, or happiness by offering courses, sometimes accredited and sometimes extra-curricular. While this is welcome, we feel that these courses miss an important opportunity. Data literacy is an important student skill set and is the ability to read, understand, create, and communicate with others using data. It encompasses an understanding of the fundamental concepts of data and statistical processing, and increasingly it includes an awareness of our own personal digital footprints (Pangrazio & Selwyn, 2019). These are the digital traces we leave behind as we use online resources and services (Haimson et al., 2016), our digital citizenship, and our cyber safety.

Most of the current generation of university students are data and information literate and (Pothier & Condon, 2020) note that companies are increasingly looking to data analytics as a core skill for all graduate recruitment and that not enough of these students have those skills. Yet, Pangrazio and Selwyn (2019) point out that there is a form of personal data literacy that is needed by all of us for us to understand and control our own personal data in contemporary society and that is the form of literacy that we address in this article. We know that students, like most others, generate and consume huge amounts of personal data and information as part of their everyday lives in their social interaction with others, their entertainment, and their education. Some of this personal data is health-related, such as data on our sleep and our exercise. Thus, it follows that we should try to use students’ own personal data, in particular personal data that is related in some way to overall wellness, as part of improving student wellness. That is because health literacy can use data literacy, and that is what we do in this article where we report on the FLOURISH module, developed and taught at our university. The closest reported work to what we report here is the work in Chisholm and Hartman-Caverly (2022) which addresses a combination of digital wellness and overall wellness goals. In this work, instructional librarians offered campus workshops for college students with an emphasis on data privacy and an awareness of our digital footprints. The workshop offered by the university library concentrated on aspects of student stress introduced by the COVID pandemic such as distancing and remote instruction rather than the wider definition of holistic wellness and well-being that we address in this article.

## Method

Work on developing a taught module in the area of student wellness and well-being started in 2020 with background work carried out by researchers which consisted of interviews, group meetings with students, student leaders, and health experts as well as further meetings with subject experts and administrative and operations staff in the University. The framework and definition for health literacy in which this was grounded were taken from Liu (2020) who defines it as the ability of an individual to find and use knowledge and information to maintain their own health. This culminated in a pilot run of a taught module during the academic year 2020/2021. From that pilot we learned the most appropriate and needed topics that should be covered and what should be the parameters for the delivery and assessment of course content. In particular, we wanted to address the question of whether harnessing personal data can support students to manage their lives and to be well, thus focusing on the interplay between health literacy and data literacy.

Working with subject experts from the university, we designed and developed a module called FLOURISH which delivers practical tips in the areas that impact students’ everyday wellness including sleep, nutrition, work habits, procrastination, relationships with others, physical activity, positive psychology, critical thinking, and more. In addition, it is also about personal data and how students can use their own personal data for their benefit. The FLOURISH module was approved by the Faculty of Engineering and Computing Teaching and Learning Committee in December 2020 as an accredited standalone module. It was offered as a module on Loop, the University’s Learning Management System (LMS). Although it was not part of any particular degree program, FLOURISH was offered as an extracurricular activity so students took FLOURISH because they wanted to, not because they needed the credit or needed to pass the module. Because FLOURISH was not formally timetabled, it was up to them to factor it into their own time schedules. The analysis work reported here was approved by University’s Data Protection Unit 07042021-DPO, and ethics approval was given by the School of Computing Research Ethics Committee. As ethical approval was deemed to be notification-only, our institutional policy is that only school-level approval was needed. Student participants provided informed consent through an informed consent form, having read a plain language statement describing the work.

FLOURISH was offered to Year 2 undergraduate students from all courses and all university campuses. It was a full-year module available entirely online where a student can gain a recognized digital certification, namely, a micro-credential worth 5 ECTS credits following its completion, if the student so chooses. According to the normal workload for the European Credit Transfer System (ECTS), 5 credits should take 125 hr of effort in total or 12 hr per topic, of which 2 to 3 hr would be viewing and reviewing online content, 2 to 3 hr would be gathering their own personal data for an assignment, 2 hr for writing and submitting an assignment, and the remainder of the time spent on reflection. These activities take place over two full semesters from mid-September to mid-May, including mid-semester and inter-semester breaks with “soft” deadlines for assessment submission. This flexibility to either complete the module and get credits or to complete part of the module and not get credits was in response to student feedback in our background research that while some students would embrace the challenge of completing the module and getting the credits, others would feel that as a form of pressure and introduce yet another stress into their lives. For such students, the flexibility to “dip into” the module and take whichever topics they wanted to or were interested in was seen as a positive aspect. Likewise, the existence of “soft” deadlines for submitting assessments was also seen as a positive, the facility to submit assignments for whichever topics a student chose, right up to the end of the academic year.

**Figure 1.**
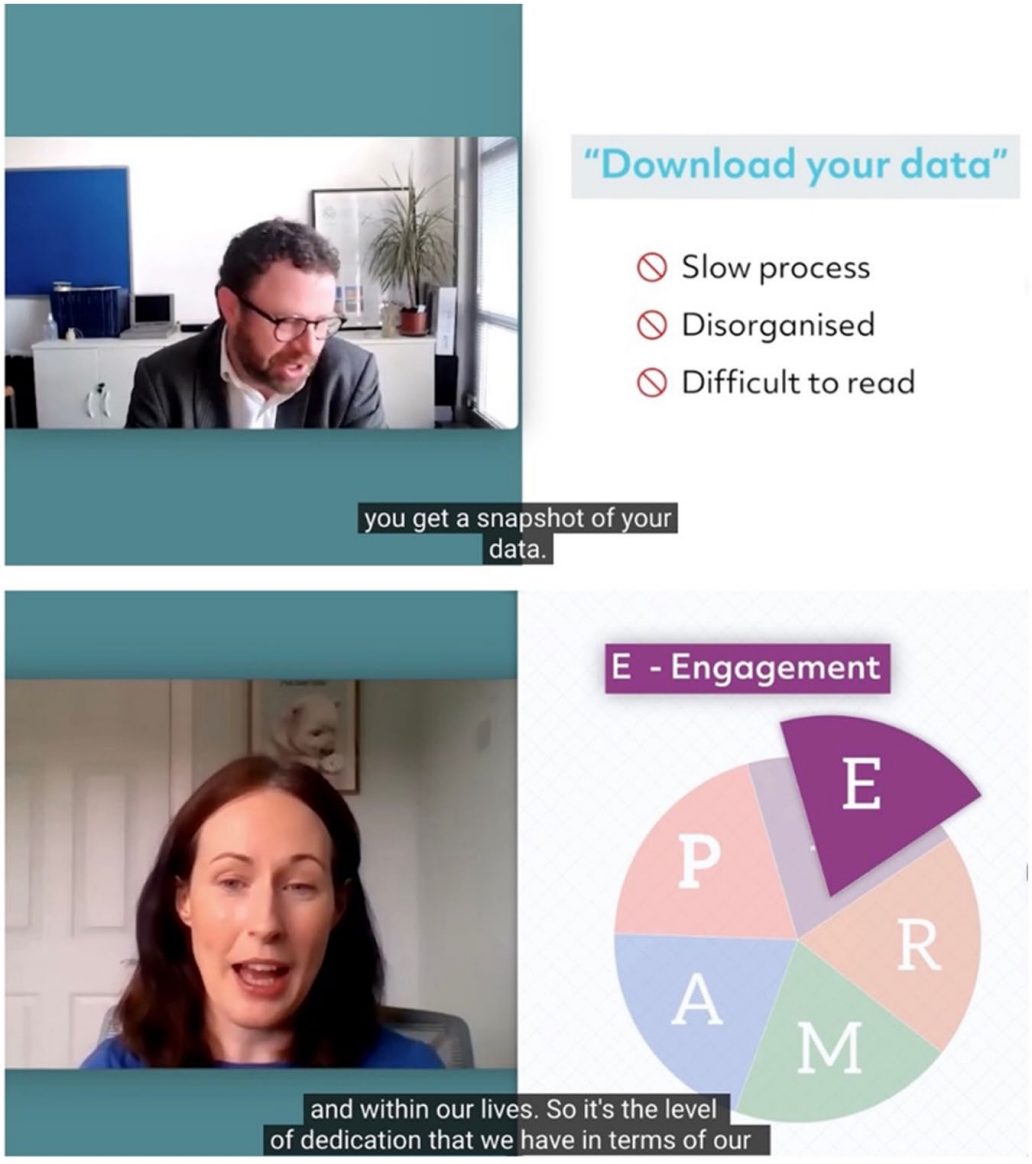
Screenshots From Video Presentations for Two of the FLOURISH Topics, What Can You Learn From Your Personal Data (Topic 1, Top), and Behavior Change (Topic 3, Bottom). Screenshots Show the Experts Being Interviewed by the Module Coordinator, the Transcript of the Spoken Dialogue and Some Visual Props on the Topics of the Dialogue.

FLOURISH content for each topic was presented online on the Loop LMS as a series of professionally produced animations and graphics, plus interview-style videos recorded between the module coordinator and almost 20 subject experts drawn from across the university. These experts included academic Faculty with clinical and research expertise in exercise science, psychology, nutrition, sleep, the use of wearable sensors and lifelogging, behavioral science, community health, organizational psychology, and work practices as well as colleagues from the University health services and the health promotion unit of these subject experts covered. This placed the module coordinator as a consistent link among the 10 topics, a familiar face carrying from one topic to the next, which is something our background work identified as being important. Also, following the feedback from our background research and experiences from the pilot program, for each topic, we created a series of video interviews between the module coordinator and the subject experts. These were a series of short-form videos of between 5 and 10 min each rather than one long video of an interview. The videos were captioned for accessibility and were professionally produced to mix shots of the topic expert and the interviewer along with animations and graphics to provide engaging content. Some of the content included questions from some students to the experts, and some screenshots illustrating this are shown in Figure 1.

The content in FLOURISH covers 10 topics and was decided by a combination of survey and overview articles in the literature including the work described in Baldwin et al. (2017), Beauchemin et al. (2018), Holman et al. (2021), Lee Wai Sum (2021), Lenstra (2020), and Ward et al. (2022), input from the subject experts in our University, and feedback from students and others obtained in the background research leading to our pilot course. In selecting the 10 topics we tried to cover as many as we could of the wellness factors described in Baldwin et al. (2017) and we have addressed the physical, social, and occupational although not the emotional or intellectual. The 10 topics are shown in Table 1 along with the learning outcomes and the assessment for each topic. These can be summarized as covering some aspects of wellness and well-being (what it is, how to change behavior, physical activity, nutrition, sleep, ways of thinking and wellbeing in work and study), some aspects of digital literacy (awareness of personal data and data footprints, gathering using your personal data), and the interplay between the two. The decision on which topics to include also considered whether or not there was an opportunity for incorporating data literacy and personal data gathering into the assignments for the topic. Some of the topic assessments namely behavior change (#3), physical activity (#4), and sleep (#6) required students to use a wearable fitness tracker to gather and analyze their own personal data on the topic. Other topics required students to use online tools including webmii.com and digi.me for learning about their personal data footprints (#1), foodbook24.com (Timon et al., 2017) in the topic on nutrition (#5) for analyzing the nutritional composition of a student’s typical diet and lumosity.com for brain training exercises where a student would directly experience hot and cold thinking in the cognitive psychology topic (#7).

**Table 1.** FLOURISH Module Topics and Assessment Details.

Number	Topic and [learning outcomes]	Assessment
1	What can you learn from your personal data ? [What size is your data footprint, how is it used]	Use the webmii.com and digi.me online tools to gather your public digital footprint and write an analysis of what you see of it.
2	What is well-being? [Domains of wellbeing, quality of life, life satisfaction]	No assessment.
3	Behavior change [Behavior change theories, effectiveness of interventions, maintenance of change]	Track your daily movement over equal periods of a baseline, days with self-monitoring and awareness, and days of self-monitoring for achieving a target. See how it varies and write an analysis of the differences.
4	Physical activity: movement [Importance of physical activity, measuring it, interventions]	Use a step counter to establish baseline steps and active exercise minutes, introduce 3 small changes to your routine and re-evaluate, then write an analysis.
5	Nutrition [Nutrition in health and its impact on wellbeing and disease causation]	Use Foodbook24, a web-based nutrition and dietary assessment tool to analyze your self-reported dietary intake, then write an analysis.
6	Sleep [Importance of sleep, sleep stages, consequences of poor sleep, sleep measurement]	Use a sleep tracker app to self-monitor your sleep for 2 weeks, introduce a change to your routine after first week and write an analysis of the impact of that routine change.
7	Cognitive psychology: ways of thinking [Executive function, attention, memory, self-control, positive emotion]	Use the Lumosity.com online tool or app to play a number of brain training games each day. Review your daily scores to see how you move from cold thinking to hot thinking, then write a reflection on your experiences with it.
8	Wellbeing in work and study [Occupational health including stress, burnout, engagement, time management]	Complete the online Utrecht Work Engagement Scale (UWES-S) and write a reflection on which of the survey questions you had a low score and what you learned from it.
9	Healthy choices [Alcohol and drugs, safe sex, smoking, reflect and evaluate your own healthy choices]	No assessment.
10	What have you learned ? [Re-evaluating your data literacy and personal digital footprint].	Sign in to your University Google account and see what Google has learned about you from your Gmail, web browsing, YouTube, Maps and other services. Then write a reflection on how you feel about Google knowing so much, whether there are errors, surprises, or omissions.

For those topics that involved the use of a wearable fitness tracker, we were influenced by the work of Kinney et al. (2019) which studied students’ use of wearable devices. That work found that among the reasons for a fitness tracker use were to monitor weight loss and to track sleep and that a common reason for their non-use was cost. To address the latter point, we distributed 130 Fitbit wearable fitness trackers among those students on the FLOURISH module who had requested one. This was funded as part of a philanthropic gift from Fitbit-Google to the university.

The topics were released online to students one at a time on the University’s LMS Loop at 2-week intervals, five in their first semester and another five in the second semester. For the eight topics that required students to either gather their own personal data (topics #1, #3, #4, #5, #6, #7, #8) or to reflect on the data their university’s Google account had gathered on them (topic #10), students were asked to write and submit a blog post on each in which they would reflect on what they had learned from the topic and to submit this for assessment. In the assessment submissions, students were encouraged to discuss whether using their own personal data on the topic had helped in providing personal insights into the topic or perhaps influencing them to change their behavior. Depending on the topic, they would use these data to reflect on some aspect of their overall wellness and to learn about themselves. This showed them how changes in their lives are reflected in their personal data, and the point of this was to help students realize that personal data can be a force for good if it is used properly and that realization is one of the learning outcomes from the module.

The availability of FLOURISH was advertised with the support of the University’s Students Union through social media, newsletters, and class lists, and as a result 169 students registered for the module in its first year. In the second year, we had even more students registered, giving us more than 350 total student registrations in 2 years. Because FLOURISH is a non-core activity, we never intended for all or even for many of the students to complete the assignments and achieve the credits. Right from their registration students were encouraged to dip into the module and take whatever topics they wanted.

## Results

At the end of the academic year when students had completed their examinations and the deadline for submitting assignments for FLOURISH had expired, we used the access log files from the Loop LMS to analyze students’ use of FLOURISH’s online resources. This is possible because the Loop LMS records every interaction with the online content by every student. Students would normally be using the Loop VLE as part of their day-to-day study for their other modules so there was no extra effort for them to connect to, and use, the FLOURISH content.

**Figure 2.**
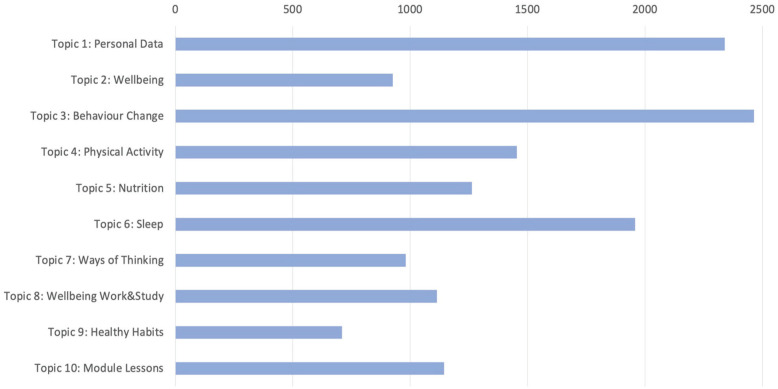
Cumulative Student Accesses to Loop Resources for Each Topic in FLOURISH.

We first examined which of the 10 topics attracted the most and least attention from students. In total, there are 73 online resources for the 10 topics on Loop, in addition to other resources such as a class noticeboard and online forum and standard the University resources on plagiarism warnings and support as well as a module descriptor. Excluding these, the cumulative student access to the resources for each topic is shown in Figure 2. This shows the topics from top to down in order of their release to students throughout the academic year. There was an average of more than 1,430 student accesses per topic from the 169 students spread across the academic year. As expected the earlier topics, 1 and 3 especially, attracted most attention because of student enthusiasm during the earlier part of the year which naturally would wane as the year progressed and as assignment deadlines and examinations loomed. The standout entry in terms of access was Topic 6 on sleep, which attracted more interest than would have been expected compared with other topics at that stage of the year. Topic 9 on Healthy Habits and to a lesser extent Topic 2 on what is wellness attracted less interest than other topics and these did not have any assessment because by their nature they did not lend themselves to gathering personal data in the same way as the other topics. The demonstrated interest in sleep and behavior change was also reflected in the number of students submitting assignments in each topic, where personal data (#1), sleep (#6), and behavior change (#3) attracted the greatest number of submitted assignments.

As the FLOURISH module ended, we conducted an anonymous survey among students asking whether FLOURISH helped to improve their overall wellness, their interaction with others, aspects of their sleep and nutrition, and whether or not FLOURISH changed their understanding of their personal data to allow them make more informed decisions about their overall wellness. The responses were an overwhelmingly strong endorsement of the impact of FLOURISH. In addition to the questions, students were invited to contribute their anonymous views on the module and these are shown in Supplemental Appendix A. Finally, when asked whether they would recommend that a student in the following year should take the FLOURISH module, 98% of those who responded replied yes.

## Discussion

In this article, we have presented the development and delivery of an accredited undergraduate module called FLOURISH which has been taken by more than 350 students from our University as an elective course. FLOURISH addresses two problems with many of our students, their health literacy and it improves their digital literacy through a raised awareness of personal data and its potential for good. Health literacy and digital literacy are each 21st-century skills heretofore not formally taught to students but learned from peers or social media, which is not a good place from which to learn life skills.

Personal data has a bad reputation (Kennedy et al., 2021) because when we hear about personal data it is almost always some breach of regulations, some leak of data, or some abuse of personal data which has negative consequences. In the case of the FLOURISH module, we do not gather students’ personal data, they gather and use it themselves and then submit assignments based on they interpreting the outcomes from the analysis of their personal data. Yet personal data can be a force for good if it is not misused or abused and in this article, we have shown how students in FLOURISH have learned to see this firsthand. Eight of the 10 topics included in the FLOURISH module have some element of personal data which students use themselves for their own benefit, whether it is learning about their own sleep, nutrition, physical activity, behavior change, or their personal digital footprints.

Based on an analysis of the use of FLOURISH’s online resources, we found that all topics were of interest but sleep and behavior change were of particular interest compared with others. This aligns with similar feedback from other courses such as that reported in White et al. (2018) which addressed behavior change in nutrition, physical activity, and mental health and found that even a brief self-care intervention in an online course showed significant improvements for students. Feedback from our students on the FLOURISH module which was year long included observations on their own behavior change, especially around nutrition and exercise, an improved life balance by taking more breaks from study and recognizing self-appreciation, a greater awareness of the importance of sleep, and an increase in their data literacy and awareness of personal data footprints.

Finally, one of the limitations of FLOURISH is that the topics tried to cover as many as we could of the wellness factors described in Baldwin et al. (2017), and we have addressed the physical, social, and occupational. However, because FLOURISH is online rather than face-to-face, it was difficult to address emotional and intellectual wellness, and we regard these aspects as out of scope for this module and requiring input from other professional sectors of the university.

The contribution that this article makes to the literature is to further endorse the need for health literacy and digital literacy to be taught in our universities, but we also demonstrate and recommend that they can be taught together with each reinforcing the other. This makes each literacy more appealing to the digital natives of Generation Z who make up the majority of our student population. The implications of this and recommendations for public health research and practice are that these two student literacies, health and digital, are not separate and should be taught together for our student cohort.

## Supplemental Material

sj-docx-1-heb-10.1177_10901981231163609 – Supplemental material for Teaching Health Literacy and Digital Literacy Together at University Level: The FLOURISH Module Click here for additional data file. Supplemental material, sj-docx-1-heb-10.1177_10901981231163609 for Teaching Health Literacy and Digital Literacy Together at University Level: The FLOURISH Module by Alan F. Smeaton in Health Education & Behavior
