# Supplementary material for: Teaching Health Literacy and Digital Literacy Together at University Level: The FLOURISH Module
Source: Health Educ Behav. 2023 Apr 18;50(5):622–8. doi: 10.1177/10901981231163609 (PMC10492429; doi:10.1177/10901981231163609)
Supplement: sj-docx-1-heb-10.1177_10901981231163609 – Supplemental material for Teaching Health Literacy and Digital Literacy Together at University Level: The FLOURISH Module [file sj-docx-1-heb-10.1177_10901981231163609.docx]

Appendix A

Having completed the module, students were asked to provide anonymous feedback on their experience, if they wished. Below is a selection of the feedback they provided.

- “The (FLOURISH) assignment was interesting to carry out and analysing my results gave me a whole new perspective on behaviour change.”
- “There are definitely things (from FLOURISH) that I would use in my life, e.g. taking breaks. Breaks are needed, especially just to clear your head a bit.”
- “I have been playing both the chalkboard challenge and memory matrix (as part of FLOURISH). Both have been really enjoyable.”
- “This (FLOURISH) topic has been an eyeopener to me and I am looking forward to using some of these tips, especially the ones relating to saving money while still eating healthy.”
- “Before I started to learn more about sleep in this (FLOURISH) topic I assumed that my sleep score just related to how long I slept ... but now I realise that REM and deep sleep play an important factor in sleep quality.”
- “This exercise was extremely interesting as I had no idea about data usage and storage before the (FLOURISH) topic.”
- “What I learned from this (step counting) is that something so passive can really improve my fitness and I would like to continue doing this and while doing so possibly manage my time better.”
- “After viewing the content in topic 1 (of FLOURISH) I was amazed at just how much of my information is being stored on different servers.”
- “Overall this (FLOURISH assignment) did increase my physical activity for the week but I would say it was more beneficial for my mental health than my physical health.”
- “The (FLOURISH assignment) has given me a new perspective on the balance between my studies and life.”
- “Due to the (FLOURISH) topic I am now doing more exercise.”
- “A great tip I learned from the videos (in FLOURISH) is simply not shopping on an empty stomach and it's so true.”
- “I found this (FLOURISH) task interesting as it gave an insight into my sleep and it explained why I may have been more tired one night than I was the other considering I had the same hours of sleep.”
- “I thought that the (FLOURISH assignments) were a nice break from the usual assignments we get in college. It allowed me to stop and think.”
- “This (FLOURISH) assignment was a great chance for me to really take note of how well I'm sleeping during an average week.”
- “I'm curious to see how the usage of data will evolve over the course of my lifetime but I will be sure to reduce how much I give to strangers now that I know how much power it gives to them.”
- “From engaging in this section of the (FLOURISH) module I have learned a lot about sleep and how it is affected by the things we do throughout the day.”
- “ (As a result of FLOURISH) I should try to build a better frame of reference for myself with regards to the work I do. I should appreciate the effort I put in ... and learn to take a breather, guilt-free.”
- “Using the new found knowledge I've discovered (from FLOURISH) I hope to be more cautious with my data going forward.”
- “I found this week's topic (in FLOURISH) to be extremely interesting. I really enjoyed playing the different games and seeing how the more I did the same game the more I improved.”
- “I noticed that the increased physical activity (from FLOURISH assignment) made me feel more awake throughout the day and made me fall asleep earlier. I also felt better about myself when I took the time out of my day to exercise.”
- “I found this part of the (FLOURISH) course to be hugely insightful and I learned a lot about myself in doing the FoodBook24 assignment, from portion sizes to the recommended calories for women.”
- “This topic (in FLOURISH) made me really think about the way I should work and take breaks.”
- “That (FLOURISH assignment) was a very interesting experience and it was interesting to see what I am actually putting into my body, do I fuel it with enough good nutrients and what things I need to cut back on. This will help me in the future and allow (me) to improve my diet.”
- “I used an app to see if my sleep quality had improved after the steps assignment but it remained unchanged, however I definitely found myself being less stressed in the evenings.”
- “I think going forward for a behavioural change for me should be enjoyable and focus on small easily achieved steps rather than setting out a large goal.”
- “Over the course of FLOURISH, I became aware of just how much information is available about me online ... I was shocked.”
- “Learning about behaviour change has been an extremely interesting and pleasant experience for me.”
